# Acute progressive stroke with middle cerebral artery occlusion caused by idiopathic hypereosinophilic syndrome: a case report

**DOI:** 10.1186/s12883-020-01941-8

**Published:** 2020-10-01

**Authors:** Quan-Fu Li, Qing Zhang, Yue-Fang Huang, Zheng-Xiang Zhang

**Affiliations:** Department of Neurology, The First Affiliated Hospital of Zhejiang Chinese Medical University (Zhejiang Provincial Hospital of Traditional Chinese Medicine), 54 Youdian Road, Hangzhou, 310006 Zhejiang Province China

**Keywords:** Idiopathic hypereosinophilic syndrome, Stroke, Middle cerebral artery, MRI

## Abstract

**Background:**

Idiopathic hypereosinophilic syndrome (IHES) is associated with various organ system dysfunctions. Neurologic abnormalities have been previously noted in this syndrome. Cerebral infarction secondary to occlusion of large cerebral artery is rarely reported. Here we described a patient with IHES presented progressive multiple cerebral infarctions caused by bilateral middle cerebral artery occlusion.

**Case presentation:**

A 55-year-old Chinese woman presented to our hospital with acute onset of right limbs weakness and slurred speech. Laboratory tests showed a significant eosinophilia of 5.29 × 10^9^/L (normal, < 0.5), 49.9% of leukocytes. Brain magnetic resonance imaging (MRI) revealed multiple acute cerebral ischemic lesions. Magnetic resonance angiography (MRA) demonstrated stenosis in horizontal segment of right middle cerebral artery. A pretibial skin biopsy revealed eosinophilic infiltration around the capillaries in deep dermis and adipose tissue. The patient was given oral dual anti platelet agents and intravenous methylprednisolone. However, one week later, the patient presented significant neurological deterioration with right-sided hemiparesis and totally motor aphasia. Brain MRI and computed tomography perfusion (CTP) demonstrated new acute cerebral ischemia in left hemisphere. Digital subtraction angiography (DSA) revealed left middle cerebral artery completely occluded. The patient received a high-dose of intravenous methylprednisolone 500 mg per day and the eosinophil count quickly fell to normal within 2 days. She was transferred to a rehabilitation center and her neurological symptoms improved with modified Ranking Scale from 4 to 2.

**Conclusions:**

IHES is one of the rare causes of acute ischemic stroke with large cerebral artery occlusion. An early high-dose of corticosteroids therapy should be considered in cases of IHES patients. Our case study is benefit to clinical diagnosis and treatment of cerebral infarction with IHES.

## Background

Idiopathic hypereosinophilic syndrome (IHES) is a rare disease characterized by an proliferation eosinophil count (greater than 1500/μL) in the peripheral blood [1]. IHES is associated with various organ system dysfunctions. In addition to cardiac and hematological pathologies, neurological alterations including encephalopathy, sensory polyneuropathy and cerebral infarction have been described [2]. However, Magnetic resonance angiography (MRA) was normal in most cases with IHES presenting ischemic stroke [3, 4]. Large vessel occlusion was rarely reported in patients with IHES. Here we report a middle aged woman presenting IHES and progressive multiple cerebral infarctions with bilateral middle cerebral artery occlusion.

## Case presentation

A 55-year-old Chinese female patient without cerebrovascular risk factors was admitted to hospital with sudden onset of right limbs weakness and slurred speech for 7 days. Her past medical history and family history were negative. She had no history of parasitic infection, allergic diseases and neoplasm. No history of fever, skin rash, joint pains, weight loss, night sweats, diarrhea was noted. She had a standard weight and free of alcohol or illegal drugs use. An initial neurologic examination revealed hemiparesis of right upper and lower extremities (4/5 strength) with extensor plantar response. Obvious pigmentation and nodules were seen in the distal skin of both legs. General medical examination results were normal. Twenty-four-hour ambulatory blood pressure monitoring and Holter recording heart’s rate/rhythm were unremarkable.

**Fig. 1:**
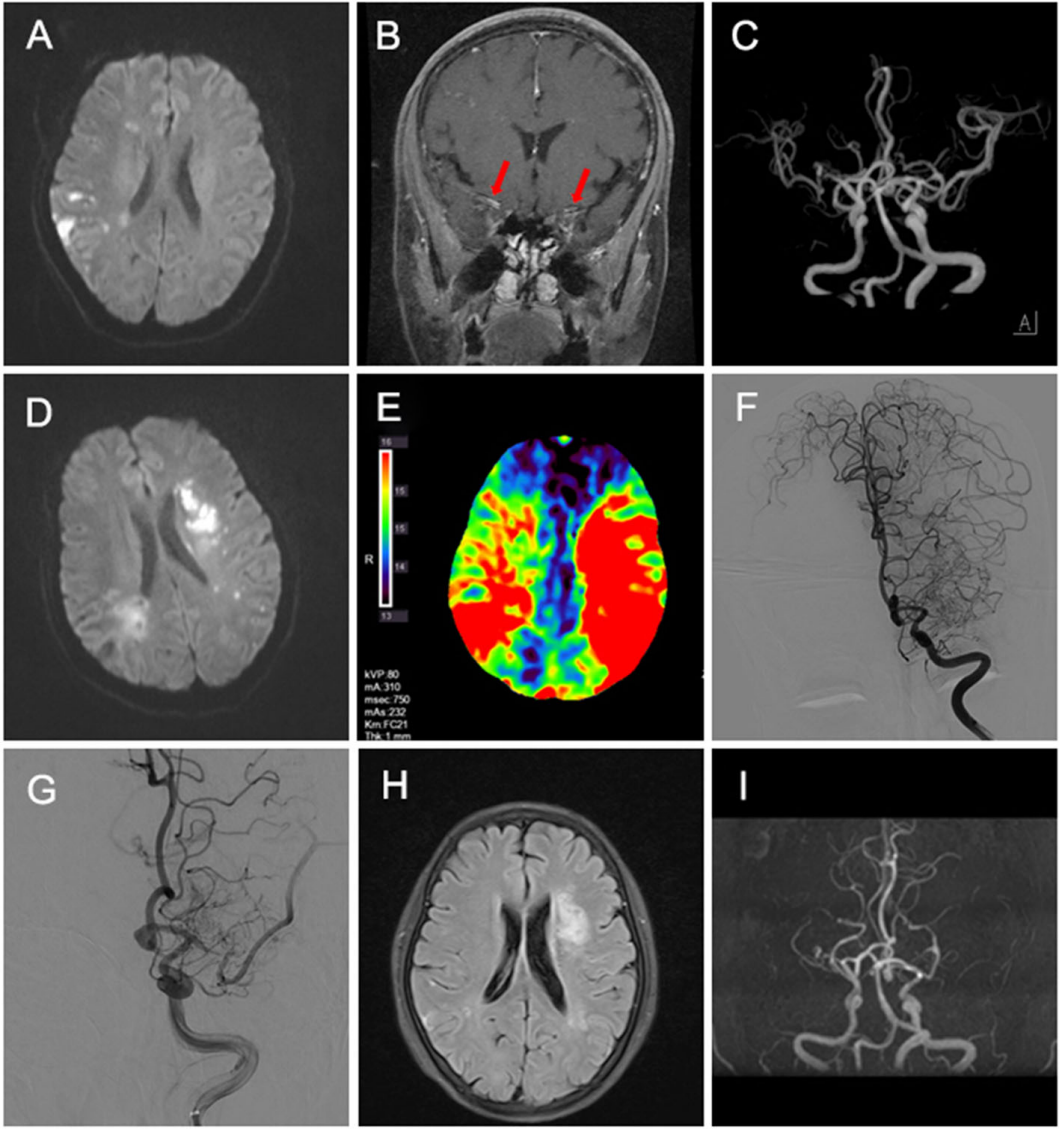
Hypereosinophilia with massive cerebral infarction and middle cerebral artery (MCA) occlusion in a 55-year-old woman. Onset Stage: **a** Diffusion-weighted magnetic resonance imaging (DWI) revealed multiple acute infarcts in right hemisphere. **b** Coronal enhanced MRI revealed bilateral middle cerebral artery wall thickening and enhancement (*red arrows*). **c** MRA showed slightly stenosis in horizontal segment of right MCA. Progression Stage: **d** DWI revealed new cerebral infarction in left hemisphere. **e** CTP showed a significantly longer peak time (TTP) in both hemispheres. **f** DSA showed M1 segment of left MCA occlusion. After endovascular therapy, partial MCA recanalization **g**. Recovery Stage: Brain flair MRI **h** demonstrated focal ischemia changes. **i** Follow-up brain MRA showed bilateral middle artery branches occlusion

**Fig. 2:**
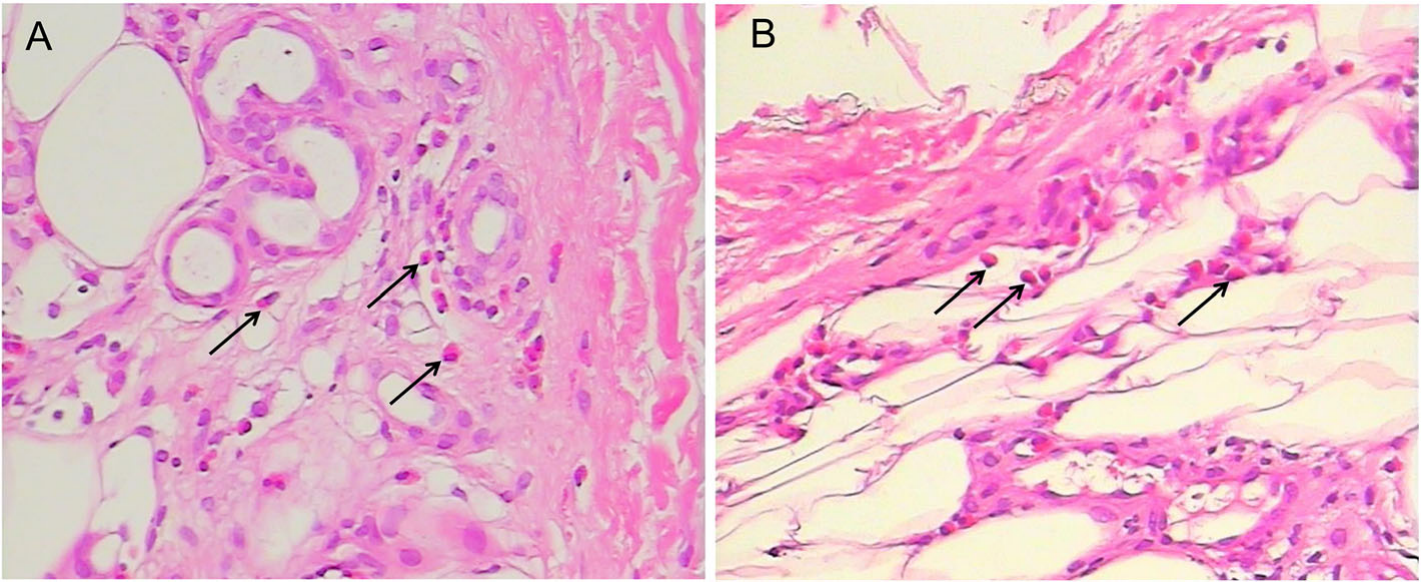
Light microscopic findings of pretibial skin. **a** Accumulation of eosinophilia around the capillaries in the deep dermis (*arrows*, H&E) . **b** Infiltration of eosinophilia in the adipose tissue (*arrows*, H&E)

Blood tests demonstrated a significant eosinophilia of 5.29 × 10^9^/L (normal, < 0.5), 49.9% of leukocytes. Autoimmune serum markers and serum parasitic antibody were all negative. No abnormal results was found in serum tumor markers, folic acid, vitamin B12, hemoglobin A1c and oral glucose tolerance test. The screen for fusion gene mutations were normal, including the platelet derived growth factor receptor (PDGFR), fibroblast growth factor receptor 1 (FGFR1), BCR-ABL and JAK2. Bone marrow puncture revealed elevated eosinophils (15%). A lumbar puncture was performed and cerebrospinal fluid protein was slightly elevated to 45.5 mg/dL (normal, < 42) with normal pressure (140 mm H_2_O) and cell count. Lung computed tomography scan, abdominal ultrasound, transesophageal echocardiography, contrast transthoracic echocardiography and contrast transcranial Doppler were normal. Brain magnetic resonance imaging (MRI) revealed multiple acute cerebral ischemic lesions (Fig. 1a). Coronal enhanced MRI revealed bilateral middle cerebral artery wall thickening and enhancement (Fig. 1b) and MRA revealed mild stenosis in horizontal segment of right middle cerebral artery (Fig. 1c). A pretibial skin biopsy revealed eosinophilic infiltration around the capillaries in deep dermis and adipose tissue (Fig. 2).

Acute ischemic stroke with IHES was diagnosed. The patient was given oral dual antiplatelet agents, aspirin 100 mg and clopidogrel 75 mg per day and intravenous methylprednisolone 40 mg per day for 5 days.

However, one week later, the patient developed wake-up stroke presenting right-sided hemiparesis (0/5 strength) and totally motor aphasia. Brain MRI and computed tomography perfusion (CTP) demonstrated new acute cerebral ischemia in left hemisphere (Fig. 1d-e). Digital subtraction angiography (DSA) was performed and M1 segment of left middle cerebral artery were completely occluded (Fig. 1f). We tried to remove the clot with support of vascular stent, but no significant blood clot was removed out. After suction, the inferior branch of left middle cerebral artery restored blood flow (Fig. 1g). Retest of peripheral blood still showed a high eosinophil count of 3.33 × 10^9^/L, 26.4% of leukocytes. Then the patient received a high-dose of intravenous methylprednisolone 500 mg per day for 4 days. The peripheral blood eosinophil count quickly fell to normal within 2 days (0.07 × 10^9^/L, 0.9% of leukocytes). She continued oral methylprednisolone 8 mg per day for maintenance.

The neurological symptoms improved slightly and she was referred to a neuro-rehabilitation hospital for treatment. With three months of rehabilitation, her muscle strength of right limbs returned to 3/5 and she could speak short or incomplete sentences. Her activity of daily living improved with modified Ranking Scale from 4 to 2. The eosinophil count remained in the normal range. However, follow-up MRA still showed bilateral middle cerebral artery branches occlusion (Fig. 1i).

## Discussion and conclusions

Eosinophilia is a hematologic condition with diverse etiologies. The underlying causes are usually classified as reactive (allergic reaction, parasitic infections or tumor), clonal (myeloid leukemia, myeloid neoplasms, or chronic eosinophilic leukemia) and idiopathic [5]. When criteria for HES are met, patients with eosinophilia of unclear cause can be diagnosed clinically as IHES. But it’s important to remember that, before making the diagnosis of IHES, all alternative diseases including reactive and clonal causes should be ruled out.

All organ systems are susceptible to the effects of eosinophilia and nearly 65% of IHES had some neurologic dysfunction [6]. Neurologic involvement in IHES patients is highly variable. Acute ischemic stroke is a common neurological complication caused by IHES. Most previously reported manifestations are mild stroke, watershed cerebral infarction, and small vascular involvement [3, 4, 7–9]. However, the patient in present study suffered from severe ischemic stroke involving bilateral middle cerebral arteries. As best as we known, there is extremely rare case report of major cerebral artery occlusion in IHES patients. Takeuchi S reported a 23-year-old female presenting acute ischemic stroke and occlusion of the M1 segment of the left middle cerebral artery resulting from HES [10]. The patient was treated with mechanical thrombolysis and partial recanalization of the left middle cerebral artery was accomplished. Raut TP described a male patient presenting with recurrent strokes over a period of three years, involvement with left and right M1 segment occlusion [11]. Compared to the two cases above, our patient presented with rapidly progressive stroke in a short period and accepted endovascular thrombectomy. Besides, our patient had poor angiogenesis and collaterals, and didn’t respond to an initial dose of corticosteroid. For patients of acute ischemic stroke with IHES, thrombolysis or thrombectomy should be administered actively within the time window. Early diagnosis and early use of corticosteroid therapy are crucial.

The mechanism of acute cerebral infarction caused by IHES remains unclear. One major theory is that it is caused by endomyocardial fibrosis and thrombosis [2]. In 52 patients with HES, six patients developed cerebrovascular thromboembolic disease and five of them had heart involvements [6]. However, others hold assumption that cerebral infarction may result from the direct cytotoxic effect of the proteins released by eosinophils and subsequent endothelial damage [12, 13]. This support the manifestations in our patient. Transesophageal echocardiography was did and no evidence of cardiac or aortic mural thrombus was found. In addition, DSA and thrombectomy were performed, which confirm that there was no obvious thrombus formation. Contrast MRI showed bilateral middle cerebral artery wall thickening and enhancement, which support cerebrovascular wall damage inflicted by eosinophilia. Herein, we speculate that vascular inflammation may be the main pathogenesis of progressive cerebral infarction in this case. Unfortunately, no cerebral vascular biopsy was obtained to confirm.

Corticosteroids have been some of the most widely utilized and most effective therapeutic agents in the treatment of IHES. The response to corticosteroid therapy is generally rapid, with reports of eosinophilia resolving within days [14]. However, our patient in present report was unresponsive to methylprednisolone of routine dose. Thus, as the cerebral infarction progressing, high dose of methylprednisolone was given and the eosinophil count quickly returned to the normal range. We recommend that hormone therapy should be administered as soon as IHES is diagnosed to reduce eosinophil levels to normal rapidly. If IHES does not respond well to standard dose of corticosteroids, a high-dose of corticosteroids therapy or other immunosuppressive drugs should be considered. Long-term treatment with low dose of steroid or other steroid-sparing medications are required to prevent relapses.

This patient illustrates multiple extensive cerebral infarction and cerebral middle artery occlusion caused by IHES. Accurate diagnosis and prompt treatment are essential. In clinical practice, IHES should also be considered when looking for the cause of cerebral infarction caused by occlusion of the major cerebral artery. Eosinophilia infiltration of cerebrovascular wall may be the main pathogenesis of cerebral infarction in this case. It also underlines the importance of early high-dose corticosteroid therapy in treatment of IHES.

## Data Availability

Data sharing is not applicable to this article as no datasets were generated or analyzed during the current study.

## Abbreviations

CTP: Computed tomography perfusion
DSA: Digital subtraction angiography
FGFR: Fibroblast growth factor receptor
IHES: Idiopathic hypereosinophilic syndrome
MRA: Magnetic resonance angiography
MRI: Magnetic resonance imaging
PDGFR: Platelet derived growth factor receptor
